# The Impact of CYP2D6 Genotyping on Tamoxifen Treatment

**DOI:** 10.3390/ph3041122

**Published:** 2010-04-15

**Authors:** Roberta Ferraldeschi, William G. Newman

**Affiliations:** 1 Department of Medical Oncology, Christie Hospital NHS Trust, Wilmslow Road, Manchester M20 4BX, UK; E-Mail: roberta.ferraldeschi@christie.nhs.uk (R.F.); 2 Genetic Medicine, St Mary's Hospital, Manchester Academic Health Sciences Centre (MAHSC), University of Manchester, Oxford Road, Manchester, M13 9WL, UK

**Keywords:** tamoxifen, *CYP2D6* genotyping, breast cancer

## Abstract

Tamoxifen remains a cornerstone of treatment for patients with oestrogen-receptor-positive breast cancer. Tamoxifen efficacy depends on the biotransformation, predominantly via the cytochrome P450 2D6 (CYP2D6) isoform, to the active metabolite endoxifen. Both genetic and environmental (drug-induced) factors may alter CYP2D6 enzyme activity directly affecting the concentrations of active tamoxifen metabolites. Several studies suggest that germline genetic variants in *CYP2D6* influence the clinical outcomes of patients treated with adjuvant tamoxifen. Here, we review the existing data relating *CYP2D6* genotypes to tamoxifen efficacy.

## 1. Introduction

The clinical benefit of tamoxifen (TAM) in the treatment of endocrine-responsive breast cancer has been evident for more than three decades. TAM is widely used for the treatment of advanced breast cancer and remains the standard adjuvant therapy for premenopausal women and a valid therapy option, alongside aromatase inhibitors (AIs) for post-menopausal women [1,2]. However, approximately one third of patients treated with adjuvant TAM have a recurrence within 15 years of surgery [3]. There is substantial inter-individual variability in response and both the beneficial and adverse effects of TAM are largely unpredictable for individual patients. 

Recent data has indicated that TAM efficacy depends on the biotransformation, predominantly via the CYP2D6 isoform, to the active metabolite endoxifen, which has greater affinity for the ER and is a more potent anti-oestrogen than the parent drug. CYP2D6 activity is extremely variable due to genetic variation and the effects of co-prescribed inhibitors of CYP2D6 [4].

Recent years have seen a number of pharmacogenetic studies evaluating the impact of *CYP2D6* germline variants on the clinical benefit of TAM adjuvant therapy. These studies have generated conflicting results. The present review aims to provide an overview on the current pharmacogenetic data and to discuss clinical implication of *CYP2D6* genotyping on TAM treatment.

## 2. *CYP2D6* Genotype and Tamoxifen Metabolism

**Figure 1:**
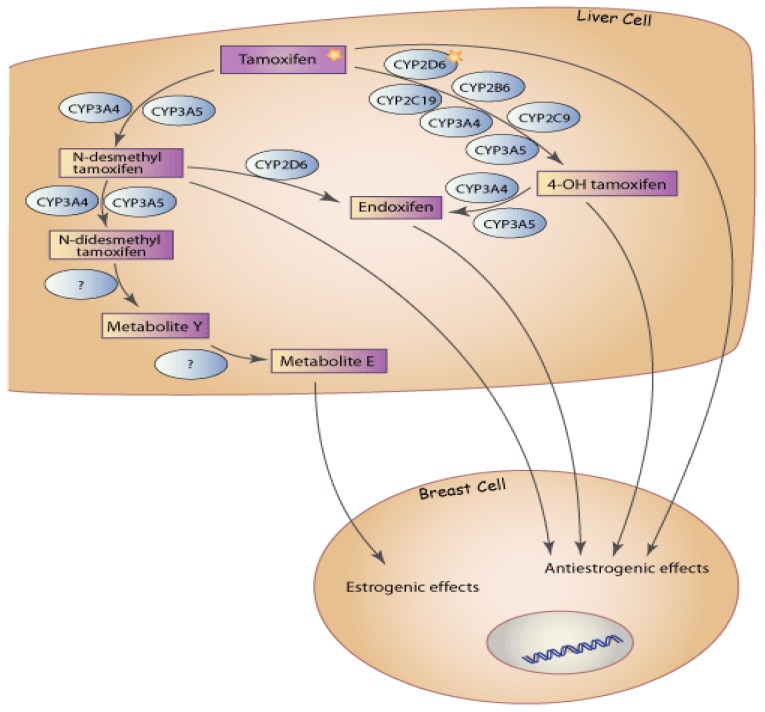
Tamoxifen metabolism in the liver. CYP3A4 and CYP3A5 are the major enzymes responsible for *N*-demethylation, whereas 4-hydroxylation is predominantly mediated by CYP2D6. Reprinted with permission. © 2009 PharmGKB. All rights reserved.

TAM is a pro-drug which is extensively metabolized in the human liver predominantly by the cytochrome P450 (CYP) system into several primary and secondary metabolites. The CYP P450-mediated biotransformation of TAM is important in determining both the clearance of the drug and its conversion to the active metabolites (Figure 1).

*N*-desmethyltamoxifen, resulting from CYP3A4/5-mediated metabolism, is the major primary metabolite, accounting for 90% of primary TAM oxidation, whereas 4-hydroxytamoxifen, resulting from CYP2D6-mediated metabolism, is a minor metabolite [5]. The latter, however, was shown to have 30- to 100-fold greater anti-oestrogen activity compared with TAM and *N*-desmethyltamoxifen [6]. *N*-desmethyltamoxifen is further oxidized to 4-hydroxy-*N*-desmethyltamoxifen (endoxifen) mainly by CYP2D6. Endoxifen is implicated as the key metabolite responsible for the effectiveness of TAM treatment, with similar potency to 4-hydroxytamoxifen in terms of suppression of oestrogen-dependent cell proliferation, ER-α and -β binding and modulation of oestrogen-mediated gene expression [7,8,9]. Further, endoxifen is normally present in up to a ten-fold greater serum concentration than 4-hydroxy-tamoxifen in women taking the standard dose of TAM [10]. Moreover, it has been proposed that endoxifen reduces ER-α protein levels by targeting it for degradation by the proteasome, while TAM and 4-hydroxytamoxifen stabilize ERα in breast cancer cells [11]. Interestingly, this effect of endoxifen has been shown to be concentration dependent and does not occur at concentrations observed in human CYP2D6 poor metabolizers.

CYP2D6 is predominantly expressed in the liver and is involved in the metabolism of many commonly prescribed drugs, including anti-depressants, anti-arrhythmics, anti-psychotics and β-blockers [12]. The *CYP2D6* gene is highly polymorphic and exhibits large inter-ethnic differences in allele frequency. Over 100 *CYP2D6* allelic variants have been described, many of which are associated with increased, decreased, or absent enzyme activity (http://www.cypalleles.ki.se). These variants result in poor (PM), intermediate (IM), extensive (EM) or ultrarapid metabolizer (UM) of CYP2D6 substrate drugs (Table 1). Among Caucasians, approximately 7–10% of individuals are PM, 10%–15% are IM, and, at the opposite end of the activity spectrum, up to 10%–15% are UM [13]. Increased activity results from gene duplication while reduced/absent activity *CYP2D6* alleles are due to deletions, stop codons or splicing defects. The most common variant alleles are CYP2D6*4 (12–21% in Caucasians), CYP2D6*5 (about 2–7% in different populations), CYP2D6*10 (>50% in Asians, 3–9% in Africans) and CYP2D6*17 (20–35% in Africans and African-Americans) [14,15,16]. Furthermore, a large number of *CYP2D6* polymorphisms with lower frequencies, but resulting in a reduced activity, contribute to the extensive inter-individual variation. CYP2D6 activity can also be inhibited by drugs, resulting in significant drug-drug interactions, that could cause therapeutic failures [17]. 

Prospective cohort studies have demonstrated that the *CYP2D6* genotype has an important role in the *in vivo* formation of endoxifen. In a study of 80 newly diagnosed breast cancer patients commencing TAM adjuvant treatment, the plasma concentrations of endoxifen after four months were lower in patients homozygous or heterozygous for non-functional *CYP2D6* alleles compared to those with two functional alleles [4]. Additionally, those subjects using potent CYP2D6 inhibitors had a 58% reduction in the plasma concentration of endoxifen. In a pharmacokinetic study of 202 Korean women who received adjuvant TAM or TAM therapy for metastatic breast cancer the investigators reported that individuals with IM alleles had endoxifen plasma concentrations two-fold lower than those of patients with normal metabolizer genotypes suggesting that the activation of TAM is also impaired in IM [18]. Additional studies have also supported the correlation between *CYP2D6* genotype and inhibitors on endoxifen plasma concentrations [19,20]. However, some variability in endoxifen plasma concentrations remained unexplained even after correction by *CYP2D6* genotype and medication history [19], notably in EM individuals endoxifen levels vary widely. 

**Table 1:** *CYP2D6* alleles and their effect on enzyme activity.

*CYP2D6* Alleles	Predicted Enzyme Activity
*1, *2, *33 and *35	Normal
*3,*4,*5,*6,*7,*8,*11-*16, *18, *19,*20,*21, *36, *38,*40, *42, *44, *56, *62, 4xn, 6xn and 36xn	None
*9,*10, *17, *29, *41, *69, 29xn and 41xn	Reduced
*22,*23-*28, *30-*32, *34,*37,*39, *43,*45-*68, *70-*75	Unknown
*1xn, *2xn,*35xn	Increased

## 3. *CYP2D6* Genotype and Tamoxifen Therapy

The first clinical evidence linking CYP2D6 metaboliser status to response to TAM was reported by Goetz *et al*. in 2005 in a subset analysis of 223 TAM-treated, postmenopausal, ER-positive patients enrolled in a randomized prospective adjuvant clinical trial (North Central Cancer Treatment Group Trial 89-30-52) [21]. Despite the inclusion of only the CYP2D6*4 and CYP2D6*6 variant alleles, which underestimates the total frequency of decreased metabolizers, the authors reported that the CYP2D6*4 allele was a predictor of a higher risk of relapse and a lower incidence of hot flashes in postmenopausal women. After a median follow-up of 11.4 years, the women homozygous for the variant genotype (CYP2D6*4/*4) had a significantly worse recurrence-free time and disease-free survival, but not overall survival compared with heterozygous and homozygous wild-type patients in univariate analyses. However, a greater proportion of women with the CYP2D6*4/*4 genotype had node-positive disease relative to that of the entire group and once nodal status and tumor size were accounted for, only a trend to significance was evident. A subsequent study on the same study population reported that co-prescription of CYP2D6 inhibitors, in addition to *CYP2D6* genetic variation, was an independent predictor of breast cancer outcome in postmenopausal women receiving TAM [43]. Results of the extended follow-up of patients in the NCCTG 89-30-52 trial presented at the San Antonio Breast Cancer Symposium in 2008, expanded the allele coverage to include the CYP2D6*3, *5, *10, *17, and *41 alleles and confirmed that PM (defined as patients with two null alleles or any patient administered a potent inhibitor) had a statistically significantly higher risk of relapse [22]. Several subsequent investigations have addressed the relationship between *CYP2D6* genotype and outcome in women treated with TAM (Table 2). Five have been consistent with Goetz’s initial findings [23,24,25,26,27]. One failed to support their findings [28], and remarkably, two others concluded the opposite effect, that PM had a better outcome on TAM [29,30] (Table 2).

Schroth *et al*. genotyped an extended number of *CYP2D6* variants including *41 and *10. Their retrospective study included 206 ER-positive patients receiving adjuvant TAM monotherapy and a control group of 280 patients not receiving TAM therapy [23]. At a median follow-up of 71 months, TAM-treated patients who carried one or two decreased/absent activity alleles, had significantly more recurrences and shorter relapse- and event-free survival compared with carriers of functional alleles. We investigated the effect of reduced *CYP2D6* genotype on the response to TAM in patients with familial early-onset breast cancer [24]. One hundred and fifteen patients with mutations in either *BRCA1* or *BRCA2*, who had been treated with adjuvant TAM (median duration of treatment >4 years) were genotyped for the CYP2D6*3, *4, *5, and *41 alleles. Combination of the individuals with two *CYP2D6* null alleles or with presumed reduced CYP2D6 activity due to concomitant use of an inhibitor defined an overall PM group, which showed a reduced time to tumor recurrence and reduced overall survival compared with all other individuals. A significant effect was only confined to patients with *BRCA2* mutations (who were more likely to have ER positive tumors) with a worse overall survival. Similarly, some other small retrospective studies reported that individuals with decreased activity *CYP2D6* variants were associated with worse outcome in patients treated with adjuvant TAM [25,26,27] (Table 2). In contrast, other groups reported no significant association or opposite results [28,29,30]. Nowell *et al*. conducted a retrospective study of 162 patients who received adjuvant TAM and 175 who did not receive hormonal therapy [28]. Subjects were genotyped for CYP2D6*3, *4 and *6 alleles. There was no significant association between the CYP2D6*4 genotype and disease recurrence or overall survival in either group of patients. In a retrospective analysis of a small subset of patients (n = 112) enrolled in a randomized clinical trial comparing postoperative radiotherapy with adjuvant chemotherapy, Wegman *et al*. reported a protective effect of the CYP2D6*4 allele [29]. In contrast to previous findings, patients treated with TAM who carried at least one CYP2D6*4 variant allele (n = 24) had better survival compared with those not treated with TAM (n = 23). Surprisingly, among patients with the *CYP2D6* wild type (normal) genotype, the outcome was similar in the TAM treated and non-TAM-treated patients. In addition, Wegman *et al*. investigated a larger retrospective cohort of 677 ER-positive, postmenopausal TAM-treated patients [30], and found patients homozygous for CYP2D6*4 had a significantly better prognosis than other patients. However, in the multivariate analysis including tumor stage, tumor size and lymph-node status, the result was less clear. Notably patients in this study received TAM at different doses and for different periods (two to five years).

Recently, a large retrospective analysis combining German and US cohorts has been reported [31]. A total of 1,361 hormone receptor positive (estrogen receptor– positive and/or progesterone receptor– positive) breast cancer patients treated with adjuvant TAM monotherapy were genotyped for CYP2D6*3, *4, *5, *10, *41 and duplications. Importantly, 350 subjects were included in previous positive association reports. Genomic DNA was extracted from different sources including blood, fresh frozen tumor and formalin-fixed, paraffin embedded tumor tissues, reflecting the different patient cohorts assembled for the study. For technical reasons, copy number variants could not be assessed in paraffin-derived samples. Compared with EM, there was a significantly increased risk of recurrence for individuals carrying one or more variant alleles. Patients lacking CYP2D6 enzyme function (PM) had an almost twofold increased risk of breast cancer recurrence compared with patients with two functional *CYP2D6* alleles (EM). Compared with EM, those with decreased CYP2D6 activity (EM/IM and PM) had worse event-free survival and disease-free survival, but genotypes did not show a significant effect on overall survival. No data were presented on treatment adherence, or regarding the co-prescription of CYP2D6 inhibitors.

We have recently undertaken a similar genotyping study of over 600 breast cancer patients, treated with TAM. We support the findings of the large German/American report with a two-fold increased risk of recurrence in patients with one or more variant alleles. The effect is most significant in post-menopausal patients treated with TAM monotherapy. Further the association is only detected when multiple *CYP2D6* variant alleles are genotyped, to ensure that patients are not misassigned to the incorrect phenotype group. Consideration of adherence to TAM in this cohort also confirmed the importance of adherence in identifying patients who are most likely to relapse [32].

The preliminary results of a large study by the International Tamoxifen Pharmacogenetics Consortium (ITPC) has been recently presented at San Antonio Breast Cancer Symposium. The ITPC was established to collect the worldwide experience relating to genetic variation in *CYP2D6* and the outcomes of women treated with adjuvant TAM. By the time of this preliminary analysis, the ITPC had received data on over 4,800 patients. However, of these only 2,880 patients were included in the initial analysis due to a number of exclusion criteria including incomplete clinical data or genotyping. The primary analysis was to determine the association of a limited range of reduced and absent activity *CYP2D6* alleles with invasive disease-free survival in TAM treated early stage, ER positive invasive breast cancer. The analysis demonstrated no association of *CYP2D6* genotype with disease-free survival or overall survival in women receiving adjuvant TAM [33]. 

## 4. *CYP2D6* Genotype in Non-Caucasian Tamoxifen Treated Breast Cancer Patients

The studies considered in the previous section have been conducted in American and European cohorts. However, as noted above, in Asia, the CYP2D6*10 allele is the major polymorphism resulting in reduced function CYP2D6. A Korean study in ER-positive metastatic breast cancer patients found that CYP2D6*10 homozygotes had lower plasma levels of TAM metabolites and a correspondingly shorter time to progression compared to patients with other *CYP2D6* genotypes [18]. In the adjuvant setting, patients homozygous for CYP2D6*10 have also been found to have significantly higher incidence of recurrence compared with wild-type individuals [34,35]. In contrast, Okishiro *et al*. did not demonstrate a significant impact of CYP2D6*10 genotype on outcome in Japanese patients with breast cancer treated with adjuvant TAM [36].

In a recent prospective study Kitoyani *et al*. investigated relationships of polymorphisms in transporter genes and *CYP2D6* to clinical outcome of patients treated with adjuvant TAM. *CYP2D6* genotyping was performed in 282 primary breast cancer patients, including 67 patients reported previously [37]. Carriage of *CYP2D6* variants was associated with shorter recurrence-free survival. Further, they confirmed that CYP2D6*10 resulted in lower plasma levels of endoxifen suggesting that the lower clinical efficacy in the TAM-treated patients with reduced CYP2D6 activity allele may be caused by lower systemic exposure to endoxifen.

## 5. Tamoxifen Side Effects and CYP2D6

Goetz *et al.* first reported that women homozygous for CYP2D6*4 had a significantly lower incidence of moderate or severe hot flashes [21]. This observation was consistent with their previous observation that CYP2D6 is responsible for the metabolic activation of TAM to endoxifen and therefore that patients with absent CYP2D6 activity would be expected to have a lower incidence of hot flashes, as the anti-oestrogen effect would be diminished. However in a small retrospective study Ramon *et al.* did not observe a significant relationship between genotype and toxicity [27]. Nevertheless, severe and mild toxicities were more frequent among PM patients than among patients with a normal metabolizing status. Rae *et al.* prospectively tested the correlation between *CYP2D6* genotypes and discontinuation rates at four months of treatment in nearly 300 TAM-treated patients [38]. Individuals were genotyped for *CYP2D6* variants and assigned a ‘score’ based on predicted allele activities. They observed a strong correlation between a higher activity score and increased rates of discontinuation. These data suggested that presence of normal *CYP2D6* alleles predict TAM discontinuation and that patients who may be most likely to benefit from TAM may paradoxically be most likely to discontinue treatment prematurely. 

**Table 2:** Summary of clinical studies that have evaluated the association between *CYP2D6* genotype and tamoxifen-related clinical outcome in early breast cancer patients.

Author	Patients	Origin of study	*CYP2D6* alleles typed	Median Follow up (years)	CYP2D6 inhibitors in PM definition	Comparison	Main Results
Goetz *et al*. 2005 [21]	N=190 ER+ve Postmenopausal TAM only	American	*4, *6	11.4	No	PM *vs*. EM + hetEM	DFS HR, 1.86; *P* = 0.089 RFS HR, 1.85; *P*= 0.176 OS HR, 1.12; *P*= 0.780
Goetz *et al.* 2007 [43]	Same as [21]	American	*4, *6	11.4	Yes	PM *vs*. EM + IM + hetEM	RFS HR, 1.74; *P* = 0.017 TTBR HR, 1.91; *P*=0.034 DFS HR, 1.60; *P*= 0.027 OS HR, 1.34; *P*= 0.223
Goetz *et al.* 2008* [22]	Same as [21]	American	*3, *4, *6, *10, *17, *41	14.5	Yes	PM *vs*. EM	TTR HR 4.0, *P*=0.001 DFS HR 2.0, *P*=0.02
Schroth *et al.* 2007 [23]	N=206 ER+ve TAM only	German	*4, *5, *10, *41	5.9	No	hetEM + IM+PM *vs.* EM	RFT HR 2.24; *P* = 0.02 EFS HR 1.89; *P* = 0.02
Newman *et al.* 2008 [24]	N=115 Familial breast cancer ER+ and -ve Adj TAM Some received CT	British	*3,*4,*5, *41	10	Yes	PM *vs.* EM+ hetEM	TTR HR, 2.1; *P* = 0.14 OS HR, 2.5; *P* = 0.17 *BRCA2* patients: TTR HR, 3.8; *P* = 0.083 OS HR, 9.7; *P* = 0.008
Bijl *et al.* 2009 [25]	N=85Adj TAM	Dutch	*4	Not available	Yes	PM *vs.* EM	BCM HR, 4.0; *P* = 0.025
Gonzalez-Santiago *et al*. 2007 * [26]	N=84 Adj TAM	Spanish	*4	5.5	No	hetEM + PM *vs.* EM	RFS HR, 2.82; *P* = 0.05
Ramon *et al.* 2009 [27]	N=91 ER+ve Adj TAM Some received CT	Spanish	Amplichip 33 alleles	9	No	*4/*4,*4/*4, *1/*5 and*2/*5 *vs.* the remaining genotypes	DFS HR not available, P = 0.016
Nowell *et al.* 2005 [28]	N=162 Adj TAM Some received CT	American	*3,*4,*6	Not available	No	PM + hetEM *vs.* EM	OS HR, 0.77; P = 0.51 PFS HR, 0.67; P= 0.19
Wegman *et al.* 2005 [29]	N=112 ER + and -ve Some received CT Some received TAM	Sweden	*4	10.7	No	Not applicable	Carriers of the CYP2D6*4 allele demonstrated a decreased risk of recurrence when treated with TAM(relative risk, 0.28; *P* =0.0089)
Wegman *et al.* 2007 [30]	N=677 ER+ve Postmenopausal Some received CT, different dose-different duration of TAM	Sweden	*4	7.3	No	PM *vs.*. EM + hetEM	RFS HR, <1; P = 0.055
Schroth *et al*. 2009 [31]	N=1325 TAM only	German / American	*3,*4,*5, *10,*41	6.3	No	hetEM+IM +PM *vs.* EM PM *vs.* EM	EFS HR, 1.33; *P*=0.01 DFS HR, 1.29; *P*=0.02 TTR HR 1.90; p=0.02
Thompson *et al.* 2009* [32]	N=618 ER+ve Adj TAM Some received CT	British	Amplichip 33 alleles	5.6	No	hetEM+ IM +PM *vs.* EM	RFS HR 1.52, *P*=0.06 Postmenopausal, TAM only patients: RFS HR, 1.96; *P*=0.036
Kitoyani *et al.* 2008 [34]	N=67 TAM only ER+ve	Japanese	*4, *5, *6, *10, *14, *18, *21, *41	8	No	IM *vs.* EM	RFS HR 10.04, *P =* 0.036
Xu *et al.* 2008 [35]	N=152 Adj TAM Some received CT No eligible women were taking CYP2D6 inhibitors	Chinese	*10	5.2	-	IM *vs.* EM + hetEM	DFS HR 4.7, *P*=0.04
Okishiro *et al* 2009 [36]	N=173 ER+ve Adj TAM Some received CT and goserelin	Japanese	*10	4.6	No	IM *vs.* EM+ hetEM	RFS HR 0.6, *P=* 0.39
Kitoyani *et al.* 2010 [37]	N=282 TAM only 67 patients reported previously [37]	Japanese	*4, *5, *6, *10, *14, *18, *21, *36, *41	7.1	No	IM +PM *vs.* EM	RFS HR 9.52, *P*=.000036

CT, chemotherapy; BCM, breast cancer mortality; BCS, breast cancer survival; CYP2D6, cytochrome P450 2D6; DFS, disease-free survival;; EFS, event-free survival; DRFS, distant recurrence-free survival; ER-+ve, oestrogen- ± progesterone-positive tumour; ER-ve, oestrogen- ± progesterone-negative tumour; hetEM – individuals with one normal *CYP2D6* allele and one null or reduced activity allele have been classified in some studies as a separate phenotype group; PFS, progression-free survival; RFS, relapse-free survival; TTBR, time to breast recurrence, TTR, time to recurrence; RFT, relapse-free time; OS, overall survival; HR, adjusted Hazard Ratio; *P*, P-value.* abstract only.

## 6. *CYP2D6* Inhibitors and Tamoxifen Therapy

Anti-depressant drugs, such as the selective serotonin reuptake inhibitors (SSRI) and the serotonin and norepinephrine reuptake inhibitors (SSNRI), are frequently used with TAM in breast cancer patients to treat depression or alleviate hot flashes [39,40,41,42]. Notably, Jin *et al*. demonstrated that the co-administration of paroxetine converts a CYP2D6 EM to a phenotypic PM, as demonstrated by a reduction in plasma endoxifen concentrations to a similar level to an individual with a PM status due to their genotype [4]. These interactions were confirmed by Borges *et al*. who reported that CYP2D6 EM who took potent CYP2D6 inhibitors had lower than expected plasma endoxifen concentrations [19]. Goetz *et al.* reported that co-prescription of CYP2D6 inhibitors was an independent predictor of breast cancer outcome in postmenopausal women receiving TAM [43]. The interaction between *CYP2D6* genotypes and the concomitant administration of an SSRI has been explored in five subsequent studies [24,25,26,32,34]. In two of these studies, CYP2D6 status contributed significantly to worse outcome [24,25]. However, recent pharmaco-epidemiological studies showed conflicting results regarding the effect of CYP2D6 inhibitors on TAM efficacy [44,45,64,65]. While uncertainty remains, potent CYP2D6 inhibitors should be avoided for the treatment of hot flashes and drugs including citalopram or venlafaxine substituted as effective alternatives [46].

## 7. Complexity of Tamoxifen Metabolism

A great deal of variability exists in steady state levels of endoxifen within the CYP2D6 EM group, suggesting that factors other than CYP2D6 influence endoxifen plasma concentrations [19]. Inter-patient variation in the activity of other polymorphic enzymes that are involved in the formation of endoxifen or in its clearance, may partly explain some of the residual variability in endoxifen concentrations. Other important cytochrome P450 isoenzymes that are involved in the bioactivation of TAM are the CYP2C9, CYP2C19, and CYP2B6 [47]. Elimination of TAM metabolites from the plasma requires the phase two drug-metabolising enzymes sulphotransferases (SULT) and UDP-glucuronosyltransferases (UGT). Sulphotransferase 1A1 is considered the primary SULT responsible for the sulphation of 4-hydroxytamoxifen and endoxifen [48,49]. Members of the UGT family, including UGT1A4, 2B15, 2B7, 1A8, and 1A10 are involved in the glucuronidation of 4-hydroxy-tamoxifen and endoxifen [50,51,52]. Genetic variants in other enzymes responsible for TAM metabolism have also been studied and recently reviewed [13,53,54]. These polymorphisms could contribute to individual variability in endoxifen plasma concentrations and patient response to TAM, and may warrant further investigation. In addition to drug-metabolizing enzymes, recent studies report that ER genotypes may further contribute to inter-individual variability to TAM [55,56].

## 8. Conclusions and Discussion

In 2005 a retrospective analysis strongly suggested that *CYP2D6* genotype was predictive of TAM efficacy [21]. These data prompted the US Food and Drug Administration (FDA) to re-label TAM to incorporate information about the genetic factors and drug interactions that may affect the efficacy of the drug. Several subsequent investigations addressing the interaction between *CYP2D6* genotype and outcomes in women who were treated with TAM in adjuvant [21,22,23,24,25,26,27,28,29,30,31,32,33,34,35,36,37], and metastatic [18] settings have been published since and reported contradictory results. Many explanations have been proposed to explain these disparate conclusions [53,57,58,59].

The first explanation that must be considered is that the association between CYP2D6 and TAM response is a false positive finding. However the functional data demonstrating the role of CYP2D6 in the conversion of TAM to endoxifen and the reduced levels of endoxifen in patients with reduced CYP2D6 activity due to inhibitor medication or genotype [4,19] is compelling and provides a rationale for the positive associations described. Discordant results may reflect the relatively small size of the some of studies, disparate patient populations, differences in dose and duration of TAM treatment, lack of information about concomitant medications and different methods and coverage of *CYP2D6* genotyping. Some of the studies have used fixed formalin paraffin embedded tissue [21,23,28] and have therefore employed genotype assays which detect only a limited number of *CYP2D6* alleles [21,22,23,24,25,26,28,29,30,31]. The method of genotyping may be critical for accurate phenotype prediction and the results of some studies of *CYP2D6* genotype may have been confounded by the inadvertent misassignment of some patients to EM. For example only few studies examined the CYP2D6*5 (*CYP2D6* gene deletion) that is present with allele frequency of 2%–7% in Caucasian individuals [15]. The lack of a comprehensive assessment of *CYP2D6* deficiency variants, may underestimate the true incidence of PM and IM.

The studies have also varied in the way that they grouped genotypes for analysis, making it difficult to compare results. Different classification of phenotype groups and comparisons between these groups (EM classifications sometimes include only patients with two functional *CYP2D6* alleles, where other classifications include individuals with one intermediate function allele) may account for further discordance. The classification of intermediate metabolizers is also complicated by consideration of whether this group should contain individuals with one absent function allele and either one normal (hetEM) or one intermediate allele. 

The use of different endpoints and the inconsistent definition of the endpoints themselves confound the interpretation of results across the studies and make cross-study comparisons difficult. Confounding arises from variation in the inclusion and exclusion of events (e.g. second non breast primary cancer, ductal carcinoma *in situ*) in definitions of end points that bear the same name (e.g. disease-free survival) [21,31] or from the use of different name to define the same clinical endpoint. For example, the disease-free survival definition in the study by Goetz *et al*. [21] corresponds to the relapse-free survival definition in the study by Schroth *et al*. [23] and to the event-free survival in the recent combined analysis of two large cohorts from USA and Germany [31]. Overall survival has been recognized as the least ambiguous and most clinically relevant clinical end point in clinical trials of cancer therapy and no compelling evidence has so far emerged that normal CYP2D6 activity improves survival in TAM treated women. The retrospective patient recruitment may have been prone to suboptimal documentation of events, insufficient control of adherence and lack of data regarding the co-prescription of CYP2D6 inhibitors. Furthermore, many of the studies may not have had sufficient power to show a difference in TAM-related efficacy between genotypes.

There are a number of potential clinical consequences from these emerging data on CYP2D6 and the outcomes of TAM treatment. TAM has been the gold standard endocrine treatment for ER-positive breast cancer for the last 25 years. Results of recent clinical trials have, however, demonstrated that third-generation AIs including anastrozole, letrozole and exemestane, are superior to TAM in the treatment of postmenopausal patients in an adjuvant or metastatic setting [60,61]. Goetz *et al.* examined a subset of postmenopausal women treated in the ABCSG-8 study, which evaluated the value of switching patients to three years of anastrazole after two years of TAM [63]. Among the patients randomized to five years of TAM, PM had a 3.8-fold increase in risk of developing breast cancer recurrence than EM in the five-year span. Interestingly, among patients who switched to anastrozole after two years of TAM, there was no increased risk of breast cancer recurrence for CYP2D6 PM in years three to five, suggesting that the benefit of switching to anastrozole, may be most pronounced in the group of patients with deficient CYP2D6 metabolism. The optimal choice for adjuvant endocrine therapy in ER-positive postmenopausal breast cancer patients has been addressed in modelling analyses [31,62]. Under the assumption that AI metabolism is independent of CYP2D6, the models suggest that among patients who are wild type for *CYP2D6* survival outcomes are similar to or perhaps even superior with TAM than with AIs. These models raise the possibility that tailored therapy based on pharmacogenomics could be considered for women who are concerned about the relative toxicity or to cut the cost of an AI as initial treatment. Final conclusions must await confirmation from large pharmacogenetic analysis in the studies comparing TAM with AIs. Ongoing pharmacogenetic analyses of materials from participants in the major randomized trials of TAM *vs*. AI (ATAC, BIG1-98, IMPACT) are largely awaited to establish the clinical utility of CYP2D6 genotyping and to determine if TAM is an equivalent, inferior or even superior therapy in patients with normal CYP2D6 activity. 

Recently, the analysis of large cohorts from the USA, Germany and UK reinforced the hypothesis that among women with breast cancer treated with TAM, there is an association between *CYP2D6* variation and clinical outcomes, such that the presence of two functional *CYP2D6* alleles is associated with better clinical outcomes [31,32]. In contrast, the ITPC analysis demonstrated no association of *CYP2D6* genotype with disease-free survival or overall survival in women receiving adjuvant TAM [33]. The ITPC represents the largest collection of patient data of *CYP2D6* genotype in adjuvant TAM therapy. However, this was only a preliminary analysis where a large number of subjects were excluded from the primary analysis because of methodological issues; incomplete information on dose and duration of TAM use; missing concomitant medication data; inadequate patient follow-up and incomplete covariate data. Within the ITPC, defining the inclusion criteria to capture the maximum available worldwide experience of CYP2D6 in tamoxifen treatment is vital whilst ensuring that this considers the most relevant clinical endpoints in a robust manner. Hence, further data collation ensuring uniformity of data quality and analyses are being undertaken by the ITPC and their results are awaited with interest.

At present, there is not enough data to justify widespread adoption of *CYP2D6* genotyping into clinical practice to guide decision-making about TAM use. Despite this we have had a number of enquiries for genotyping to direct prescription and both clinicians and patients are aware of this emerging data. Formal recommendations on the integration of *CYP2D6* genotypes into treatment decisions must await further data from larger retrospective analyses or prospective studies. Additional adequately powered studies are also needed to address some unresolved issues. Few data are available on pre-menopausal women and the impact of chemotherapy or subsequent AI treatment and none of the studies has investigated the associations between *CYP2D6* genotype, endoxifen levels and treatment outcome. Prospective studies are underway to address these important issues. In the interim, we await the results of the analyses of the companion pharmacogenetic studies on the randomized controlled trials of TAM *versus* AIs as these will provide the most robust determination of the value of *CYP2D6* genotype in tailored adjuvant treatment. 
